# Response to “Avoiding Methodological Bias in Studies of Amyloid Imaging Results Disclosure”

**DOI:** 10.1186/s13195-019-0504-1

**Published:** 2019-06-04

**Authors:** Joshua D. Grill, Chelsea G. Cox, Kristin Harkins, Jason Karlawish

**Affiliations:** 1 Institute for Memory Impairments and Neurological Disorders, Irvine, CA USA; 2 Institute for Clinical and Translational Science, Irvine, CA USA; 3 Department of Psychiatry and Human Behavior, Irvine, CA USA; 4 Department of Neurobiology and Behavior, University of California, Irvine, CA USA; 5 Penn Memory Center, Philadelphia, PA USA; 6 Department of Medicine, Philadelphia, PA USA; 7 Department of Medical Ethics and Health Policy, Philadelphia, PA USA; 8 Department of Neurology, University of Pennsylvania, Philadelphia, PA USA

The goal of “Reactions to learning a ‘not elevated’ amyloid PET result in a preclinical Alzheimer’s disease trial” was to study how learning one is not eligible for a trial based on an Alzheimer’s disease (AD) biomarker result affects willingness to be in subsequent trials, as well as how it affects other behaviors [1]. Answering this question fills a critical gap in the literature, as preclinical AD trials are increasingly common but the ideal criteria for participant inclusion remains an area of active research. Thus, a person ineligible for one trial may be eligible for another.

Taswell and colleagues correctly observe that our study did not include a comparison group, which would have necessarily been individuals who demonstrated elevated amyloid and therefore were eligible for randomization in the preclinical AD trial [2]. The primary question under study—whether a subject who screen-fails for one AD trial is willing to participate in subsequent trials—was not applicable to this population. Their exclusion is not a bias. It was not necessary.

Looking more broadly, the gist of Taswell and colleagues’ commentary is a sensible summary of social science research. Multiple methods, and indeed multiple studies, are needed to arrive at a common understanding of the way the world is and how it can be changed. Only then can high confidence be achieved. In the nascent space of preclinical AD trials, much remains to be learned and a breadth of research designs and methods will be essential to advancing the field.
